# A 10- and 15-year performance analysis of ESC/EAS and ACC/AHA cardiovascular risk scores in a Southern European cohort

**DOI:** 10.1186/s12872-020-01574-2

**Published:** 2020-06-19

**Authors:** Cátia Santos-Ferreira, Rui Baptista, Manuel Oliveira-Santos, José Pereira Moura, Lino Gonçalves

**Affiliations:** 1 Cardiology Unit, Centro Hospitalar e Universitário de Coimbra, Coimbra, Portugal; 2 Coimbra Institute for Clinical and Biomedical Research (iCBR), Faculty of Medicine, University of Coimbra, Coimbra, Portugal; 3 Internal Medicine Unit, Centro Hospitalar e Universitário de Coimbra, Coimbra, Portugal; 4 Faculty of Medicine, University of Coimbra, Coimbra, Portugal

**Keywords:** Atherosclerosis, Cardiovascular risk, Guidelines, Lipids, Myocardial infarction, Stroke

## Abstract

**Background:**

A key strategy for the primary prevention of cardiovascular disease (CVD) is the use of risk prediction algorithms. We aimed to investigate the predictive ability of SCORE (Systematic COronary Risk Estimation) and PCE (Pooled Cohort Equations) systems for atherosclerotic CVD (ASCVD) risk in Portugal, a low CVD risk country, at the 10-year landmark and at a longer, 15-year follow-up.

**Methods:**

The SCORE and PCE 10-year risk estimates were calculated for 455 and 448 patients, respectively. Discrimination was assessed by Harrell’s C-statistic. Calibration was analyzed by standardized incidence ratios (SIR).

**Results:**

During the 10-year follow-up, 7 fatal ASCVD events (the SCORE outcome) and 32 any ASCVD events (the PCE outcome) occurred. The SCORE system showed good discrimination (C-statistic 0.83), while the PCE showed poor discrimination (C-statistic 0.62). Calibration was similar for both systems, according to SIR: SCORE, 0.3 (95% CI 0.1–0.7); PCE, 0.5 (95% CI 0.4–0.7). Globally, both 10-year fatal ASCVD risk and any ASCVD risk were overestimated in the overall population and men. However, the risk was underestimated by both systems in women. Despite an overestimation of 15-year fatal ASCVD by SCORE, the 15-year any ASCVD observed incidence was 1.8 times the 10-year incidence among men and 1.4 times among women. This acceleration of CVD risk was more relevant in the lowest classes of ASCVD risk.

**Conclusion:**

In this prospective, contemporary, Portuguese cohort, the SCORE had better discriminatory power and similar calibration compared to PCE. However, both risk scores underestimated 10-year ASCVD risk in women.

## Background

Cardiovascular disease (CVD) remains the leading cause of morbidity and mortality in developed countries, despite consistent improvement in outcomes [1]. Modifiable risk factors are the major causes of atherosclerotic CVD (ASCVD), accounting for 90% of cardiovascular risk [2, 3]. A key strategy in the primary prevention of ASCVD is the use of risk prediction algorithms, as the prophylactic interventions should target the people who should benefit from them most [1, 4]. However, it is debatable which is the best algorithm for ASCVD risk estimation.

In 2003, the European Society of Cardiology (ESC) jointly with the European Atherosclerosis Society (EAS) published the SCORE (Systematic COronary Risk Estimation): a 10-year risk estimation system for ACSVD death [5]. The SCORE Project pooled data from 12 European prospective cohort studies from eleven countries, including Southern European countries such as Spain and Italy, but not Portugal [5]. External validation has been performed for the SCORE model in several Western countries and in the Asian population [6–10]. However, calibration of the risk charts according to cardiovascular mortality levels of each country has been proposed for a more precise prediction of risk estimates [5]. This is of utmost importance in countries not represented in the derivation cohorts, as Portugal.

In parallel, the American College of Cardiology (ACC) and American Heart Association (AHA) developed the Pooled Cohort Equations (PCE) that estimate a composite endpoint of 10-year ASCVD risk, instead of the SCORE-estimated 10-year fatal ASCVD risk [11]. However, these equations were all derived from North American cohorts [11], which limit their applicability to other populations. Further, the PCE have been controversial because of reports that they substantially overestimate risk in both American and European populations [12–16].

Besides the different prediction models available to estimate the ASCVD risk, the decision thresholds recommended for drug therapy are different for, respectively, fatal ASCVD (SCORE) and any ASCVD (PCE) [17]. Such conflicting recommendations may create confusion among physicians, potentially reflecting uncertainty about the external validity of different algorithms under different settings [18], namely for low-risk countries, like those in Southern Europe [19, 20]. In Portugal, a country with one of the lowest ASCVD event rates in Europe [20], neither risk systems have been validated and doubts concerning their clinical applicability and predictive accuracy in this particular population are raised.

In this study, we aimed to determine the 10- and 15-year incidence of ASCVD events in a primary prevention cohort of a Southern European country. In addition, we aimed to compare the calibration and discrimination of both SCORE and PCE systems in this cohort at the predefined 10-year landmark and at a longer, 15-year follow-up, in order to estimate their external validity for clinical practice.

## Methods

### Study design and population

We conducted a prospective single-center study, including 663 patients consecutively referred by Primary Care physicians to *Centro Hospitalar e Universitário de Coimbra*’s Lipidology Clinic from 1994 to 2007. All patients were followed until December 2017. ﻿Among recruited participants, we selected those aged 40–79 years and with no previous history of heart disease and stroke at the screening visit. Patients with familial hypercholesterolemia or with missing information at the baseline examination were excluded, resulting in 455 individuals available for this study.

### Assessment of risk factors

Baseline, demographic and clinical variables were collected, including age at referral, sex, hypertension, diabetes and smoking status. Hypertension was defined as systolic blood pressure ≥ 140 mmHg and/or diastolic blood pressure ≥ 90 mmHg, or current antihypertensive treatment. Diabetes was defined as fasting glucose ≥126 mg.dL^− 1^ or the use of hypoglycemic drugs. Smoking status was dichotomized as current versus past/never smokers. On the first appointment, several baseline laboratory variables and a complete lipid profile on fasting blood samples were collected.

### Risk classification definitions

The predictors used to estimate risk for a first fatal or any ASCVD event included age, sex, smoking status, total cholesterol (TC), high-density lipoprotein cholesterol (HDL-C), systolic blood pressure, diabetes, and antihypertensive treatment. SCORE and PCE systems vary in the age ranges to which they apply. SCORE is recommended for use in the 40- to 65- year age range [1]. In contrast, the age range in which PCE is validated is 40–79 years [11]. Thus, we compared the performance of both risk systems in individuals aged 40–79 years. Baseline SCORE (using the model for low-risk countries) [5] and PCE [11] predicted risks were calculated for each patient. To comply with real-life clinical use of the SCORE, the absolute fatal ASCVD risk for those aged 66–79 years was corresponding to the risk at age 65 [21]. The cohort was stratified in four groups based on the SCORE risk categories: low (SCORE < 1%), moderate (SCORE 1–5%), high (SCORE 5–10% or diabetes without organ damage or major CVD risk factor), and very high (SCORE > 10% or diabetes with organ damage or major CVD risk factor) [1]. Major CVD risk factors were defined by markedly elevated single risk factor, namely TC > 310 mg.dL^− 1^ and blood pressure ≥ 180/110 mmHg [1]. Regarding the PCE risk, the cohort was also stratified in four risk categories: low (ASCVD risk < 5%), borderline (ASCVD risk 5–7.5%), intermediate (ASCVD risk 7.5–20%), and high (ASCVD risk > 20%) [4].

### Outcome variables

In SCORE, the predicted outcome is fatal ASCVD, comprising the occurrence of death from coronary artery disease, stroke, hypertension, heart failure, peripheral artery disease or aortic disease [5]. The endpoint defined by PCE is a first ASCVD event, including nonfatal myocardial infarction, ASCVD death, and stroke [11]. The events among the study cohort were identified through record linkage with the national health registry and Health Data Platform. We ascertained nonfatal myocardial infarction or stroke events, defined according to the Cardiovascular and Stroke Endpoint Definitions for Clinical Trials [22]. Cases of fatal ASCVD [22] were ascertained from the cause of death listed on death certificates.

### Statistical analysis

A total of 455 patients had complete information on the components of SCORE and 448 had complete information on the components of PCE. The median (interquartile range (IQR)) follow-up time was 15 (11–17) years. All patients were followed up for at least 10 years and 55.4% (*n* = 252) were followed for 15 years regarding fatal ASCVD and 48.9% (*n* = 219) regarding any ASCVD.

Continuous variables were expressed as mean ± standard deviation (SD). Median and IQR were used if the distribution was not normal, assessed by the use of the Kolmogorov-Smirnov test. The Student’s t-test for normal variables and the Mann-Whitney test for non-normal variables were used for comparisons among groups (male vs female). Categorical variables were presented as percentages and were compared using chi-square or Fisher’s exact test.

The predictive ability of the SCORE and PCE systems for the Southern European population was evaluated based on discrimination and calibration of the models. The discriminative power was compared with Harrell’s C-statistics, which takes into account the timing of events. A calibration analysis was conducted by using standardized incidence ratios (SIR), comparing the 10-year predicted event rate to the observed rate at 10-year follow-up. As all patients completed the 10-year follow-up, 10-year observed event rate was the actual rate. Regarding the calibration analysis at the 15-year follow-up, the Kaplan–Meier method was utilized to estimate the 15-year observed risk for each group. Both rates were plotted against the predicted risk, estimated as mean 10-year risk score in each group [23]. Participants were censored at the time of the first occurrence of ASCVD, death, time of the last follow-up, or at 15 years of follow-up.

Concerning head-to-head comparison, an intersection of the two risk systems specific subgroups was created (*n* = 448). The discrimination was evaluated by Harrell’s C-statistic for each risk system-specific outcome. The pairwise differences among the C-statistics corresponding to the risk systems were calculated, and, to compensate for multiple testing, bootstrapping was used to obtain 99% confidence intervals (CI) [23].

All tests were conducted using STATA 13.0 (StataCorp, Texas, USA).

## Results

﻿The mean age ± SD of the study sample of 455 individuals was 57.8 ± 9.6 years and 61.8% were male. The overall baseline characteristics were similar between genders, except that women had significantly higher levels of low-density lipoprotein cholesterol (LDL-C) and a larger proportion of statin therapy (Table 1).

**Table 1:** Baseline characteristics and observed events in a Portuguese cohort aged 40–79 years in primary prevention

	Total (*n* = 455)	Male (*n* = 281)	Female (*n* = 174)	*P* value
**Age – years**	57.8 ± 9.6	57.8 ± 9.5	57.8 ± 9.7	0.44
**BMI – kg.m**^**−2**^	27.8 (25.8–30.8)	28.1 (25.9–30.2)	27.5 (25.2–31.8)	0.36
**Systolic BP – mmHg**	135 (125–150)	135 (125–150)	135 (125–150)	0.95
**Diastolic BP – mmHg**	85 (80–90)	85 (80–90)	85 (80–90)	0.98
**Antihypertensive drugs – no. (%)**	260 (57.1)	156 (55.5)	104 (59.8)	0.37
**Creatinine – mg.dL**^**−1**^	0.9 (0.8–1.1)	0.9 (0.8–1.1)	0.9 (0.8–1.1)	0.15
**Total cholesterol – mg.dL**^**−1**^	268.0 (226.5–309.0)	263.0 (222.0–306.0)	273.0 (231.0–316.0)	0.05
**HDL-C – mg.dL**^**−1**^	46.0 (38.0–58.0)	46.0 (38.0–57.0)	47.0 (39.0–59.5)	0.21
**LDL-C – mg.dL**^**−1**^	158.4 ± 57.8	151.8 ± 56.6	170.4 ± 57.9	0.02
**Statin treatment - no. (%)**	298 (65.5)	173 (61.6)	125 (71.8)	0.03
**Diabetes – no. (%)**	76 (16.7)	49 (17.4)	27 (15.5)	0.59
**Current smokers – no. (%)**	85 (18.7)	51 (18.1)	34 (19.5)	0.71
**Mean calculated risk**				
**SCORE - %**	4.7 ± 6.8	5.6 ± 7.9	3.1 ± 4.0	< 0.001
**PCE risk - %**	13.5 ± 12.5	16.2 ± 13.7	9.0 ± 8.3	< 0.001
**Median calculated risk**				
**SCORE - %**	2.7 (0.7–6.1)	3.6 (1.5–7.5)	1.0 (0.3–5.0)	< 0.001
**PCE risk - %**	9.5 (4.5–18.3)	12.8 (6.6–22.1)	6.1 (3.1–11.9)	< 0.001
**Fatal ASCVD**				
**10-year – no. (%)**	7 (1.6)	2 (0.7)	5 (2.9)	0.11
**15-year – no. (%)**	5 (1.9)	1 (0.7)	4 (3.7)	0.15
**Any ASCVD**				
**10-year – no. (%)**	32 (7.0)	15 (5.4)	17 (9.8)	0.07
**15-year – no. (%)**	25 (11.3)	12 (9.8)	13 (13.5)	0.71

Continuous variables were expressed as mean ± SD. Median and IQR were used if the distribution was not normal, assessed by the use of the Kolmogorov-Smirnov test. The Student’s t-test for normal variables and the Mann-Whitney test for non-normal variables were used for comparisons among groups. Categorical variables were presented as percentages and were compared using chi-square or Fisher’s exact test. 15-year fatal and any ASCVD were Kaplan–Meier adjusted

*ASCVD* Atherosclerotic cardiovascular disease, *BMI* Body mass index, *BP* Blood pressure, *HDL-C* High density lipoprotein cholesterol, *IQR* interquartile range, *LDL-C* Low-density lipoprotein cholesterol, *PCE* Pooled Cohort Equations, *SCORE* Systematic COronary Risk Estimation, *SD* standard deviation

The average 10-year risk estimates by each of the risk systems were as follows: the mean risk for ASCVD mortality was 4.7% (5.6% in men and 3.1% in women) according to SCORE and the mean ASCVD risk was 13.5% (16.2% in men and 9.0% in women) according to PCE, globally indicating a population with moderate ASCVD risk (Table 1).

### Discrimination

We assessed the ability of SCORE and PCE to discriminate between patients who developed events defined by SCORE (fatal ASCVD) and PCE (any ASCVD) and those who did not, after calculating the 10-year risk for each patient. During the 10-year follow-up, 7 (1.6%) SCORE-specific events and 32 (7.0%) PCE-specific events occurred (Table 1). The C-statistic corresponding to the model with risk score as the only covariate were 0.83 and 0.62 for the SCORE-specific and PCE-specific outcomes, respectively (Table 2).

**Table 2:** Harrell’s C-statistic SCORE and PCE in predicting fatal ASCVD events and any ASCVD events in a Portuguese cohort aged 40–79 years in primary prevention, respectively

	**OVERALL**	**Men**	**Women**
C_SCORE_ (fatal ASCVD)			
10-year (n = 455)	0.83 (0.74–0.93)	0.87 (0.69–1.00)	0.87 (0.78–0.96)
15-year (n = 252)	0.80 (0.70–0.90)	0.86 (0.67–1.00)	0.84 (0.75–0.93)
C_PCE_ (any ASCVD)			
10-year (n = 448)	0.62 (0.53–0.71)	0.66 (0.52–0.79)	0.67 (0.56–0.79)
15-year (n = 219)	0.63 (0.55–0.70)	0.64 (0.53–0.75)	0.67 (0.57–0.78)

The predictive ability of the risk scores was assessed by Harrell’s C-statistic for risk system-specific outcomes

*ASCVD* Atherosclerotic cardiovascular disease, *PCE* Pooled Cohort Equations, *SCORE* Systematic COronary Risk Estimation

Regarding the 15-year follow-up, 5 (1.9%) ASCVD deaths and 25 (11.3%) any ASCVD events were observed. The C-statistic were 0.80 for the SCORE-specific outcomes and 0.63 for PCE-specific outcomes (Table 2).

**Fig. 1:**
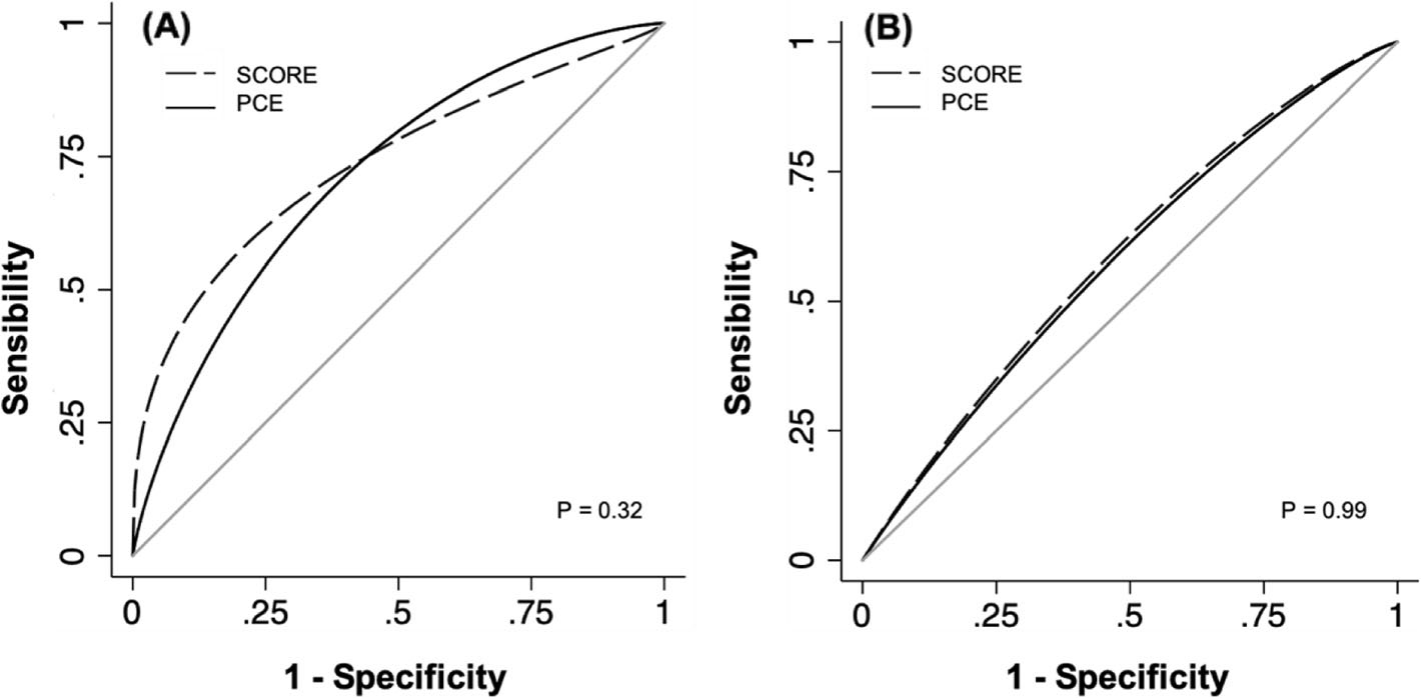
ROC curves of the SCORE and PCE models for prediction of 10-year events in a Portuguese cohort aged 40–79 years in primary prevention. **a** Fatal ASCVD events and **b** any ASCVD events. The predictive ability of the risk scores was assessed by Harrell’s C-statistic for risk system-specific outcomes. ASCVD: atherosclerotic cardiovascular disease, SCORE: Systematic COronary Risk Evaluation, PCE: pooled cohort equations

In the head-to-head comparison, while the SCORE displayed the highest C-statistics for fatal ASCVD, the values were similar considering any ASCVD as the outcome (Fig. 1). No statistically significant differences were observed between the predictive ability of the two risk systems (Table 3).

**Table 3:** Head to head comparison of the risk scores according to Harrell’s C-statistic in a Portuguese cohort aged 40–79 years in primary prevention (*n* = 448)

		**C**_**SCORE**_	**C**_**PCE**_	**△(C**_**SCORE**_**-C**_**PCE**_) **(99% CI)**	***P*value**
Fatal ASCVD					
n = 7	10-YEAR	0.83	0.78	0.05 (−0.05–0.15)	0.32
*n* = 5	15- YEAR	0.80	0.75	0.05 (− 0.04–0.15)	0.27
Any ASCVD					
*n* = 32	10- YEAR	0.62	0.62	0.00 (−0.06–0.06)	0.99
n = 25	15- YEAR	0.63	0.62	0.00 (−0.06–0.04)	0.72

The predictive ability of the risk scores was assessed by Harrell’s C-statistic for each risk system-specific outcome. The pairwise differences among the C-statistics corresponding to the risk systems were calculated, and, to compensate for multiple testing, bootstrapping was used to obtain 99% confidence intervals (CI)

*ASCVD* Atherosclerotic cardiovascular disease, *CI* Confidence interval, *PCE* Pooled Cohort Equations, *SCORE* Systematic COronary Risk Estimation

### Calibration

#### Score

**Fig. 2:**
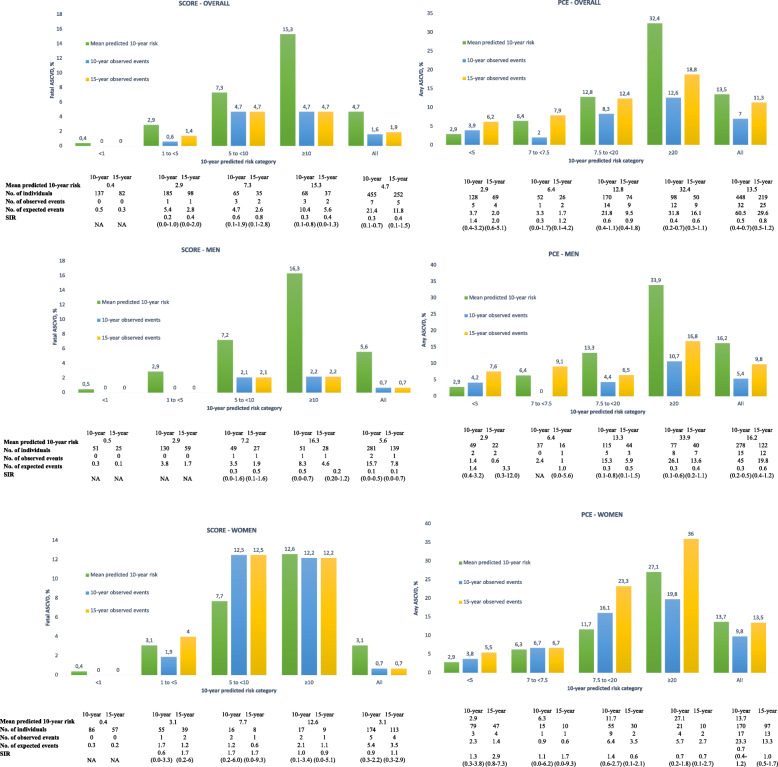
Calibration accuracy evaluated by comparing observed and predicted events and standardised incidence ratios in a Portuguese cohort aged 40–79 years in primary prevention. A calibration analysis was conducted by using SIR, comparing the 10-year predicted event rate to the observed rate at 10- and 15-year follow-up. As all patients completed the 10-year follow-up, 10-year observed event rate was the actual rate. 15-year observed events were Kaplan–Meier adjusted. SIR, standardised incidence ratios, SCORE: Systematic COronary Risk Evaluation; PCE: pooled cohort equations

While 7 fatal events occurred during the 10-year follow-up period, SCORE predicted 21.4 events, which means there were 66% more expected events than observed (Fig. 2).

Discordance between observed and expected cases was found throughout the risk continuum, with a predominance of overestimation in the overall population and men by all of the risk strata at 10-year and 15-year follow-up. Importantly, SCORE underestimated risk in women, particularly in those at high risk (5 to 10%) (Fig. 2).

#### PCE

Globally, the PCE predicted 47% more any ASCVD events (Fig. 2). Actually, the PCE overestimated 10-year risk when the predicted risk was 5% or higher. Both in the overall population and men, when the predicted risk was low (< 5%), the risk was slightly underestimated. Regarding women, PCE underestimated risk in low (< 5%) and intermediate (7.5–20%) risk strata. In the 219 individuals with 15 years of follow-up, an acceleration of the risk was observed, surpassing the 10-year predicted risk in low (< 5%) and borderline (5–7.5%) risk strata in men and low (< 5%) and intermediate (7.5–20%) risk strata in women (Fig. 2).

## Discussion

In our cohort of Portuguese patients, the evaluation of PCE and SCORE risk prediction algorithms revealed that both performed at an acceptable level. The SCORE system performed better than the PCE system in discriminating their respective endpoints. Although calibration was similar, both risk systems markedly overestimated the risk.

﻿While both risk systems address the same practical question, which individuals benefit from interventive measures, its equations differ significantly. The SCORE includes as predictors age, sex, smoking, systolic blood pressure, and TC; it is traditionally applicable in individuals from 40 to 65 years, and the predicted outcome is 10-year fatal ASCVD, disregarding non-fatal events [5]. Besides the predictors included in SCORE, PCE also incorporates ethnicity, HDL-C, antihypertensive use, and diabetes. Furthermore, PCE has a larger age range (40–79 years) and predicts the 10-year risk of fatal and non-fatal ASCVD [11].

Existing studies regarding the SCORE risk prediction system generally reported good discrimination, consistent with our findings [5, 8, 9, 24]. Considering the SCORE system, our study reported a favorable C-statistic of 0.83. The discrimination performance of the SCORE risk to predict 10-year ASCVD mortality was evaluated in the SCORE project and the C-statistic values ranged from 0.71 to 0.84 [5]. In the global population, we observed a systematic risk overestimation that has also been previously reported in low-risk populations, as in Spain [8, 9], and high-risk populations [24].

The external validation studies that evaluate the PCE system have generated controversy around the predictive accuracy of these equations in contemporary cohorts [25]. The PCE models discriminated risk reasonably (C-statistic 0.62) in our cohort, but performing worse in comparison to other studies [11, 15, 16]. The original PCE C-statistic ranged from 0.71 to 0.82 [11] and 0.74 to 0.79 in contemporary cohorts [16]. Similar to the SCORE, we observed a risk overestimation in our population, with an exception in predicting ASCVD risk in low-risk strata (< 5%). Previous literature established that the PCE generally overestimate risk among modern European and American populations [13, 15, 16, 23, 26]. Although there are no reports of underestimation of ASCVD risk by the PCE, it has been widely recognized that a large number of people with ASCVD risk prediction < 7.5% will paradoxically experience ASCVD events [27], reinforcing the value of additional risk markers to reclassify those patients [4, 27].

Accurate calibration of the risk prediction models is crucial for the success of preventive measures. Although both models overestimated the risk, when observed risks were taken into account, the accuracy of risk prediction in women was less impressive. Actually, the risk was underestimated by the PCE risk system in the low-risk (< 5%) and intermediate-risk (7.5–20%) strata. Underestimation of ASCVD risk in the female gender has only been described once previously in a Korean cohort [28].

Concerning the SCORE system, the interaction between sex and events should be analyzed as an exploratory and hypothesis-generating finding, due to the very low number of 10-year fatal ASCVD events (*n* = 7), especially in men (*n* = 2). This low ASCVDmortality rate is consistent with what has been found in a Spanish cohort of 608 non-diabetic patients on primary prevention with a similar moderate 10-year risk of fatal ASCVD (2.1%), where only 9 ASCVD deaths were reported (1.5%) [8]. Nevertheless, women’s risk was underestimated in high-risk women (5–10%). An underestimation of the risk by the SCORE system in women has already been reported in Australian women below 50 years [6], and in a Malaysian population [7]. Conversely, in another Southern European cohort from Italy, the 10-year fatal ASCVD risk was properly predicted by SCORE [29]. However, the low-risk (< 1%) women from this cohort had a 20-year rate of any ASCVD of 3.7, and 40% of ASCVD events occurred in this class, which signals potential problems with the calibration of this equation in women [29].

The causes of the unexpected findings regarding the underprediction of fatal or any ASCVD risk in women and the very low number of ASCVD deaths in men are not clear. Although women from our cohort had higher levels of LDL-C, they were also treated more commonly with statins. In the past, numerous studies have shown excess mortality in women after myocardial infarction [30]. In addition, women suffering an acute coronary syndrome are treated less intensively than men [31], and, even on primary prevention, it has been observed a lower use of lipid-lowering drugs in women [32]. Independently of the cause of the excess risk of ASCVD events in women, our results suggest that at least in our population, the risk stratification systems might need to be better calibrated among women, in order to reduce the proportion of women miscategorized and to avoid CVD events by implementing appropriate preventive measures [5].

Lastly, the SCORE overestimated the 15-year risk in the overall population, probably reflecting the decline of CVD mortality in the last decades [33]. Conversely, regarding PCE, we observed an important increase in any ASCVD events at 15-year follow-up. The observed 15-year any ASCVD incidence was 1.8 times the 10-year incidence among men and 1.4 times among women. This acceleration of ASCVD risk is even more relevant in the low- and borderline-risk strata in men and the low- and intermediate-risk strata in women, as the observed 15-year risk surpasses the 10-year predicted risk. A divergence between short-time and long-term risk has already been described in two Italian studies [29, 34]. Among the relatively large group with low and intermediate short-term risk, differences in risk factor burden seem to translate into marked differences in the incidence of ASCVD events in the remaining lifespan [35]. The added value of long-term risk estimation becomes even more relevant in women and younger men, more prone to be classified as being at low short-term risk [36].

﻿It is essential to evaluate the applicability of risk models to each population, as risk scores may perform worse in a different setting from the one they were originally derived [37]. It is well-known that the incidence of CVD events is declining, and Portugal is no exception. Despite the high prevalence of hypertension and other chronic diseases, Portugal has one of the best indicators for cardiovascular mortality [20], namely age- and risk-standardized mortality rates concerning coronary artery disease and cerebrovascular disease [19]. This problem is well-recognized and the literature recommends that scores should be recalibrated if incidence rates differ substantially in the new settings [5, 13], as we have previously discussed for women. Additionally, newer statistical methods could considerably improve the accuracy of ASCVD risk estimates [16]. Another, more straightforward option to improve its predictive ability would be the incorporation of additional risk variables such as ﻿coronary artery calcium score and high-sensitivity C-reactive protein [4, 27] or ﻿consider the lifetime risk for CVD in addition to short-time (10-year) risk [4].

We acknowledge several limitations of our study. We have a limited sample size, representative of the Center region of Portugal, one of the regions with the lowest rate of CVD events in our country [20]. This may explain partially the overestimation of the risk by both systems, and extrapolation of our results to other populations should be done cautiously. In addition, only half of the patients had a complete 15-year follow-up, as many patients were included in this cohort after 2003. Also, we cannot overlook that two-thirds of our cohort was under statin therapy; however, the majority of the patients were under low- and moderate- intensity statins as they were recruited before the era of high-intensity statins recommendation and are representative of a real-world population seen in everyday practice. Interestingly, in a sensitivity analysis of the Copenhagen General Population Study, including statin users at baseline (7% of the population) did not explain the observed mismatch between the predicted and observed event rates [21]. Finally, we were also not able to adjust for prescription of other preventive medication during follow-up. This might also have contributed to some of the observed mismatch. A strength of our study is that the results originate from the largest contemporary Portuguese cohort evaluated so far, with no patient lost to follow-up.

## Conclusions

In this prospective, contemporary, Portuguese cohort, SCORE had better discriminatory power compared with PCE. Overall, both systems overestimated ASCVD risk at the 10-year landmark. However, among women, ASCVD risk was underestimated with both risk systems. Lastly, we also observed an acceleration of the cardiovascular risk at 15-year follow-up, particularly at the lowest risk classes. These findings may have the potential to improve risk stratification and, ultimately, treatment allocation in a primary prevention setting.

## Data Availability

The datasets used and/or analyzed during the current study are available from the corresponding author on reasonable request.

## Abbreviations

ACC: American college of cardiology
AHA: American Heart Association
ASCVD: Atherosclerotic cardiovascular disease
CVD: Cardiovascular disease
EAS: European atherosclerosis society
ESC: European society of cardiology
HDL-C: High-density lipoprotein cholesterol
LDL-C: Low-density lipoprotein cholesterol
PCE: Pooled cohort Eqs.
TC: Total cholesterol
SCORE: Systematic COronary risk estimation
SD: Standard deviation
SIR: Standardized incidence ratios
